# Complete Genome Sequences of Thermus thermophilus Strains HB5002 and HB5008, Isolated from Mine Hot Spring in Japan

**DOI:** 10.1128/MRA.00272-21

**Published:** 2021-04-22

**Authors:** Kentaro Miyazaki, Toshiyuki Moriya, Natsuko Tokito, Tairo Oshima, Kei Yura, Yoshitaka Bessho

**Affiliations:** a Bioproduction Research Institute, National Institute of Advanced Industrial Science and Technology (AIST), Tsukuba, Ibaraki, Japan; b Department of Computational Biology and Medical Sciences, Graduate School of Frontier Sciences, The University of Tokyo, Kashiwa, Chiba, Japan; c Institute of Environmental Microbiology, Kyowa-kako Co. Ltd., Machida, Tokyo, Japan; d Graduate School of Humanities and Sciences, Ochanomizu University, Bunkyo, Tokyo, Japan; e Center for Interdisciplinary AI and Data Science, Ochanomizu University, Bunkyo, Tokyo, Japan; f School of Advanced Science and Engineering, Waseda University, Shinjuku, Tokyo, Japan; g RIKEN SPring-8 Center, Sayo, Hyogo, Japan; h Institute of Biological Chemistry, Academia Sinica, Taipei, Taiwan; Georgia Institute of Technology

## Abstract

We isolated Thermus thermophilus strains HB5002 and HB5008 from Mine Hot Spring in Japan. Whole-genome sequencing revealed that they showed ∼100% average nucleotide identity to each other, ≥98.53% to the T. thermophilus strains originating from the same spot but ≤97.64% to the T. thermophilus strains from geographically different places in Japan.

## Abstract (precis)

We isolated Thermus thermophilus strains HB5002 and HB5008 from Mine Hot Spring in Japan. Whole-genome sequencing revealed that they showed ∼100% average nucleotide identity to each other, ≥98.53% to the T. thermophilus strains originating from the same spot, but ≤97.64% to the T. thermophilus strains from geographically different places in Japan.

## ANNOUNCEMENT

Thermus thermophilus was first isolated from Mine Hot Spring in Japan in 1968 (1). Since then, many T. thermophilus strains have been isolated from various thermal areas worldwide (2–6). The thermophile is aerobic and grows optimally at around 70°C, with a doubling time of ∼1 h; basic genetic engineering techniques were developed in the mid-1980s (7). These favorable properties pushed the species as a model thermophilic organism, and researchers have intensively studied T. thermophilus biochemically (8, 9), structurally (10, 11), and genetically (12–14). As a 50th anniversary project, we initiated an ecological investigation of the thermophile—how T. thermophilus strains thrive/survive in Mine Hot Spring. In 2018, we collected a water sample from exactly the same fountain geyser at Mine Hot Spring from which the representative strains HB8 and HB27 were isolated (1). Dozens of T. thermophilus strains were isolated (6, 15) and preliminarily classified into several groups based on appearance—color, colony morphology, growth rate, and so on. In this study, we conducted whole-genome analyses of strains HB5002 and HB5008 by combining Oxford Nanopore Technologies (ONT) and Illumina technologies.

Cells were grown at 70°C in Lennox LB medium, and genomic DNA was purified using a blood and cell culture DNA midikit (Qiagen). For long-read sequencing, unsheared genomic DNA was pretreated with a short-read eliminator kit (Circulomics) to remove <10-kbp fragments, and a library was constructed using a ligation sequencing kit (ONT). Sequencing was performed with a GridION X5 system on a FLO-MIN106 R9.41 flow cell (ONT). Base calling was conducted using Guppy v.4.0.11 to generate 156,751 reads with an average length of 7,040 bases (total, 1.10 Gb) for HB5002 and 28,202 reads with an average length of 11,561 bases (total, 326 Mb) for HB5008. For all software, default parameters were used unless otherwise noted. The raw sequencing data were filtered (Q < 10; length, <1,000 bases) using NanoFilt v.2.3.0 (16), yielding 122,280 reads (longest read, 241,328 bases; *N*_50_, 15,542 bases; total, 977 Mb) for HB5002 and 21,082 reads (longest read, 149,286 bases; *N*_50_, 23,988 bases; total, 276 Mb) for HB5008. For short-read sequencing, a Nextera DNA Flex library prep kit (Illumina) was used to generate libraries with an ∼700-bp insert. Sequencing was performed on a MiSeq instrument (Illumina), yielding 1,746,904 (HB5002) and 945,019 (HB5008) paired-end reads (2 × 256 bases). The raw sequencing data were filtered (Q < 30; length, <10 bases) using fastp v.0.20.1 (17), yielding 1,407,029 paired-end reads (average length, 225 bases; total, 634 Mb) for HB5002 and 724,958 paired-end reads (average length, 224 bases; total 325 Mb) for HB5008.

The trimmed long- and short-read data were assembled using Unicycler v.0.4.8 (18) and polished using Pilon v.1.23 (19), generating a single circular chromosome and three circular plasmids for HB5002 and a single circular chromosome and two circular plasmids for HB5008. Rotation and circularity were confirmed via Unicycler. Automatic annotation was conducted using DFAST v.1.2.4 (20), and the genomic features are summarized in Table 1. FastANI analysis (21) indicated that the genome sequences of HB5002 and HB5008 showed ∼100% average nucleotide identity (ANI) to each other, ≥98.53% ANI to those of the strains originating from Mine Hot Spring (HB8, GenBank accession number NC_006461.1; HB27, NC_005835.1 [22]; HB5018, NZ_AP024270 [15]; HC11; NZ_AP019801 [6]) but ≤97.64% ANI to those of the strains originating in Arima Hot Spring (AA2-20, NZ_AP019792.1; AA2-29, NZ_AP019794.1) (5). The results mirrored habitat-specific genomic conservation.

**TABLE 1:** Genome statistics and features of Thermus thermophilus strains HB5002 and HB5008

Strain	Chromosome or plasmid	Length (bp)	GC content (%)	No. of CDSs^*a*^	No. of rRNAs	No. of tRNAs	GenBank accession no.
HB5002	Chromosome	2,042,948	69.5	2,196	6	54	AP024301
	Plasmid, pHB5002b	102,370	68.4	115	0	0	AP024302
	Plasmid, pHB5002c	12,913	69.0	6	0	0	AP024303
	Plasmid, pHB5002d	7,983	69.4	15	0	0	AP024304
HB5008	Chromosome	2,041,893	69.5	2,194	6	54	AP024305
	Plasmid, pHB5008b	102,367	68.4	115	0	0	AP024306
	Plasmid, pHB5008c	7,984	69.4	15	0	0	AP024307

a CDSs, coding DNA sequences.

### Data availability.

The complete genome sequences of T. thermophilus HB5002 and HB5008 are available from DDBJ/EMBL/GenBank under the accession numbers summarized in Table 1. The raw sequencing data were deposited in the SRA database under the accession numbers DRA011332 (HB5002) and DRA011333 (HB5008).

## References

[1] Oshima T, Imahori K. 1974. Description of *Thermus thermophilus* (Yoshida and Oshima) comb. nov., a nonsporulating thermophilic bacterium from a Japanese thermal spa. Int J Sys Evol Microbiol 24:102–112. doi:10.1099/00207713-24-1-102.

[2] Manaia CM, Hoste B, Gutierrez MC, Gillis M, Ventosa A, Kersters K, Da Costa MS. 1995. Halotolerant *Thermus* strains from marine and terrestrial hot springs belong to *Thermus thermophilus* (ex Oshima and Imahori, 1974) nom. rev. emend. Syst Appl Microbiol 17:526–532. doi:10.1016/S0723-2020(11)80072-X.

[3] Santos MA, Williams RAD, Da Costa MS. 1989. Numerical taxonomy of *Thermus* isolates from hot springs in Portugal. Syst Appl Microbiol 12:310–315. doi:10.1016/S0723-2020(89)80079-7.

[4] Fujino Y, Kawatsu R, Inagaki F, Umeda A, Yokoyama T, Okaue Y, Iwai S, Ogata S, Ohshima T, Doi K. 2008. *Thermus thermophilus* TMY isolated from silica scale taken from a geothermal power plant. J Appl Microbiol 104:70–78. doi:10.1111/j.1365-2672.2007.03528.x. 17850299

[5] Miyazaki K, Tomariguchi N. 2019. Complete genome sequences of *Thermus thermophilus* strains AA2-20 and AA2-29, isolated from Arima Onsen in Japan. Microbiol Resour Announc 8:e00820-19. doi:10.1128/MRA.00820-19. 31371550 PMC6675998

[6] Miyazaki K. 2019. Complete genome sequencing of *Thermus thermophilus* strain HC11, isolated from Mine Geyser in Japan. Microbiol Resour Announc 8:e00873-19. doi:10.1128/MRA.00873-19. 31558631 PMC6763646

[7] Koyama Y, Hoshino T, Tomizuka N, Furukawa K. 1986. Genetic transformation of the extreme thermophile *Thermus thermophilus* and of other *Thermus* spp. J Bacteriol 166:338–340. doi:10.1128/jb.166.1.338-340.1986. 3957870 PMC214599

[8] Yamada T, Akutsu N, Miyazaki K, Kakinuma K, Yoshida M, Oshima T. 1990. Purification, catalytic properties, and thermal stability of threo-Ds-3-isopropylmalate dehydrogenase coded by *leuB* gene from an extreme thermophile, *Thermus thermophilus* strain HB8. J Biochem 108:449–456. doi:10.1093/oxfordjournals.jbchem.a123220. 2277037

[9] Miyazaki K. 2005. A hyperthermophilic laccase from *Thermus thermophilus* HB27. Extremophiles 9:415–425. doi:10.1007/s00792-005-0458-z. 15999224

[10] Brodersen DE, Clemons WM, Jr, Carter AP, Wimberly BT, Ramakrishnan V. 2002. Crystal structure of the 30 S ribosomal subunit from *Thermus thermophilus*: structure of the proteins and their interactions with 16 S RNA. J Mol Biol 316:725–768. doi:10.1006/jmbi.2001.5359. 11866529

[11] Yokoyama S, Hirota H, Kigawa T, Yabuki T, Shirouzu M, Terada T, Ito Y, Matsuo Y, Kuroda Y, Nishimura Y, Kyogoku Y, Miki K, Masui R, Kuramitsu S. 2000. Structural genomics projects in Japan. Nat Struct Biol 7:943–945. doi:10.1038/80712. 11103994

[12] Tamakoshi M, Uchida M, Tanabe K, Fukuyama S, Yamagishi A, Oshima T. 1997. A new *Thermus*-*Escherichia coli* shuttle integration vector system. J Bacteriol 179:4811–4814. doi:10.1128/jb.179.15.4811-4814.1997. 9244269 PMC179328

[13] Hoseki J, Yano T, Koyama Y, Kuramitsu S, Kagamiyama H. 1999. Directed evolution of thermostable kanamycin-resistance gene: a convenient selection marker for *Thermus thermophilus*. J Biochem 126:951–956. doi:10.1093/oxfordjournals.jbchem.a022539. 10544290

[14] Miyazaki K, Tomariguchi N. 2019. Occurrence of randomly recombined functional 16S rRNA genes in *Thermus thermophilus* suggests genetic interoperability and promiscuity of bacterial 16S rRNAs. Sci Rep 9:11233. doi:10.1038/s41598-019-47807-z. 31375780 PMC6677816

[15] Miyazaki K, Moriya T, Nemoto N, Oshima T, Yura K, Bessho Y. 2021. Complete genome sequence of *Thermus thermophilus* strain HB5018, isolated from Mine Hot Spring in Japan. Microbiol Resour Announc 10:e00039-21. doi:10.1128/MRA.00039-21. 33707321 PMC7953284

[16] De Coster W, D'Hert S, Schultz DT, Cruts M, Van Broeckhoven C. 2018. NanoPack: visualizing and processing long-read sequencing data. Bioinformatics 34:2666–2669. doi:10.1093/bioinformatics/bty149. 29547981 PMC6061794

[17] Chen S, Zhou Y, Chen Y, Gu J. 2018. fastp: an ultra-fast all-in-one FASTQ preprocessor. Bioinformatics 34:i884–i890. doi:10.1093/bioinformatics/bty560. 30423086 PMC6129281

[18] Wick RR, Judd LM, Gorrie CL, Holt KE. 2017. Unicycler: resolving bacterial genome assemblies from short and long sequencing reads. PLoS Comput Biol 13:e1005595. doi:10.1371/journal.pcbi.1005595. 28594827 PMC5481147

[19] Walker BJ, Abeel T, Shea T, Priest M, Abouelliel A, Sakthikumar S, Cuomo CA, Zeng Q, Wortman J, Young SK, Earl AM. 2014. Pilon: an integrated tool for comprehensive microbial variant detection and genome assembly improvement. PLoS One 9:e112963. doi:10.1371/journal.pone.0112963. 25409509 PMC4237348

[20] Tanizawa Y, Fujisawa T, Kaminuma E, Nakamura Y, Arita M. 2016. DFAST and DAGA: Web-based integrated genome annotation tools and resources. Biosci Microbiota Food Health 35:173–184. doi:10.12938/bmfh.16-003. 27867804 PMC5107635

[21] Jain C, Rodriguez-R LM, Phillippy AM, Konstantinidis KT, Aluru S. 2018. High throughput ANI analysis of 90K prokaryotic genomes reveals clear species boundaries. Nat Commun 9:5114. doi:10.1038/s41467-018-07641-9. 30504855 PMC6269478

[22] Henne A, Bruggemann H, Raasch C, Wiezer A, Hartsch T, Liesegang H, Johann A, Lienard T, Gohl O, Martinez-Arias R, Jacobi C, Starkuviene V, Schlenczeck S, Dencker S, Huber R, Klenk H-P, Kramer W, Merkl R, Gottschalk G, Fritz H-J. 2004. The genome sequence of the extreme thermophile *Thermus thermophilus*. Nat Biotechnol 22:547–553. doi:10.1038/nbt956. 15064768

